# Dl-3-*n*-Butylphthalide Exerts Dopaminergic Neuroprotection Through Inhibition of Neuroinflammation

**DOI:** 10.3389/fnagi.2019.00044

**Published:** 2019-02-28

**Authors:** Yajing Chen, Tingting Wu, Heng Li, Xuan Li, Qing Li, Xiaoying Zhu, Mei Yu, Sheng-Han Kuo, Fang Huang, Yun-Cheng Wu

**Affiliations:** ^1^Department of Neurology, Shanghai General Hospital, Shanghai Jiao Tong University School of Medicine, Shanghai, China; ^2^Department of Neurology, Jinan Central Hospital Affiliated to Shandong University, Jinan, China; ^3^The State Key Laboratory of Medical Neurobiology, The Institutes of Brain Science and the Collaborative Innovation Center for Brain Science, Shanghai Medical College, Fudan University, Shanghai, China; ^4^Department of Neurology, College of Physicians and Surgeons, Columbia University, New York, NY, United States

**Keywords:** dl-3-*n*-butylphthalide, MAPK, microglia, neuroinflammation, NF-κB, Parkinson’s disease

## Abstract

Microglia-mediated neuroinflammation contributes to multiple neurodegenerative disorders, including PD. Therefore, the regulation of microglial activation probably has the therapeutic potential. This study is aimed to determine whether NBP could suppress microglial activation and protect dopaminergic neurons from excessive neuroinflammation. In the present study, MPTP-induced PD model was established to explore the neuroprotective and anti-inflammatory effect of NBP. We assessed motor deficits, dopaminergic neurodegeneration and microglial activation in PD mice. *In vitro*, the anti-inflammatory activity of NBP was confirmed by cell viability assay of SH-SY5Y cells after being treated with conditioned medium from LPS-stimulated BV-2 cells and from 1-Methyl-4-phenylpyridinium iodide (MPP^+^)-stimulated BV-2 cells. The expression of pro-inflammatory molecules was determined by RT-PCR, Western Blot and ELISA assay. The generation of NO and ROS were also assessed. The involvement of signaling pathways such as MAPK, NF-κB, and PI3k/Akt were further investigated by Western Blot and immunofluorescence assay. The neuroprotective effect of NBP was demonstrated *in vivo* as shown by the improvement of dopaminergic neurodegeneration, motor deficits and microglial activation in MPTP-induced mouse model of PD. The expression of pro-inflammatory mediators was also reduced by NBP administration. *In vitro*, NBP also protected dopaminergic neurons from neurotoxicity induced by activated microglia. NBP pretreatment not only reduced pro-inflammatory molecules, but also suppressed NO release and ROS generation in BV-2 cells. Further mechanism research suggested that the inactivation of MAPK, NF-κB and PI3K/Akt may involve in anti-neuroinflammation role of NBP. In conclusion, our results revealed that NBP exerted dopaminergic neuroprotection through inhibition of microglia-mediated neuroinflammation, suggesting the promising therapeutic effect of NBP for PD.

## Introduction

Parkinson’s disease is the second most common neurodegenerative disorder, and its typical clinical manifestation are static tremor, bradykinesia, rigidity and abnormal posture (Przedborski, 2017). Although the main neuropathological characteristics of PD have been well-established as progressive loss of nigral dopaminergic neurons with formation of α-synuclein-containing Lewy bodies, the mechanism of PD occurrence and progression still remains unclear. DA replacement therapy is hardly satisfying since it only provides partial symptomatic relief and can barely slow the progression of dopaminergic neurodegeneration. Abundant neuroprotective drugs which were proven effective in experimental PD models turned out failure in clinical trials (Athauda and Foltynie, 2015). Therefore, it is urgent to identify the promising agents for arresting PD progression.

Neuroinflammation is characterized by the activation of glial cells, including both microglia and astrocyte, accompanied with the increase of pro-inflammatory molecules such as IL-1β, TNF-α, iNOS, COX-2 and so on (Ransohoff, 2016). Evidence for activation of microglia in PD has been addressed using various methods. The biopsy of PD patients showed the accumulation of activated microglia in the striatum and SN (Damier et al., 1999). The ongoing activation of microglia in PD patients was confirmed by positron emission tomography imaging (Edison et al., 2013; Stokholm et al., 2017). The level of microglial activation was found to be associated with clinical severity of PD patients (Croisier et al., 2005; Stokholm et al., 2017). A meta-analysis of anti-inflammatory drug trials also revealed a significant relationship between the use of non-aspirin non-steroidal anti-inflammatory drugs and the reduced risk for developing PD, indicating the contribution of neuroinflammation to the disease (Gagne and Power, 2010). Hence, the inhibition of microglial activation and neuroinflammation has a strong therapeutic potential for PD.

Dl-3-*n*-butylphthalide is racemization form of l-3-*n*-butylphthalide and is primarily used to treat acute ischemic patients in clinical practice (Xue et al., 2016; Yan H. et al., 2017; Zhang et al., 2017). Accumulating evidence demonstrated that NBP also produced protective effects in neurodegenerative diseases including Alzheimer’s disease, PD and amyotrophic lateral sclerosis (Feng et al., 2012; Xiong et al., 2012; Wang et al., 2016). In a rotenone induced PD model, NBP exerted neuroprotective effects against oxidative damage and neuronal injuries through its antioxidant property (Xiong et al., 2012). An *in vitro* study showed that NBP protected PC12 cells against neurotoxicity by inducing autophagy-mediated α-synuclein degradation (Liu et al., 2012). In addition to antioxidant property and autophagic mechanism, recent researches have suggested a neuroprotection role of NBP via inhibiting microglial activation in rats following traumatic spinal cord injury (He et al., 2017) and in mice injected with LPS (Zhao et al., 2016; Chen et al., 2018). It is unclear whether NBP could play a beneficial role in MPTP-treated mice, a commonly used experimental PD model, and how NBP influence microglia activation in the nigrostriatal dopaminergic system.

In the present study, we investigated the neuroprotective and anti-inflammatory effect of NBP in PD with MPTP-induced PD mouse model and further explored the anti-inflammatory mechanism in LPS-activated and MPP^+^-activated BV-2 microglia *in vitro*.

## Materials and Methods

### Animals and Drug Treatments

**Figure 1:**
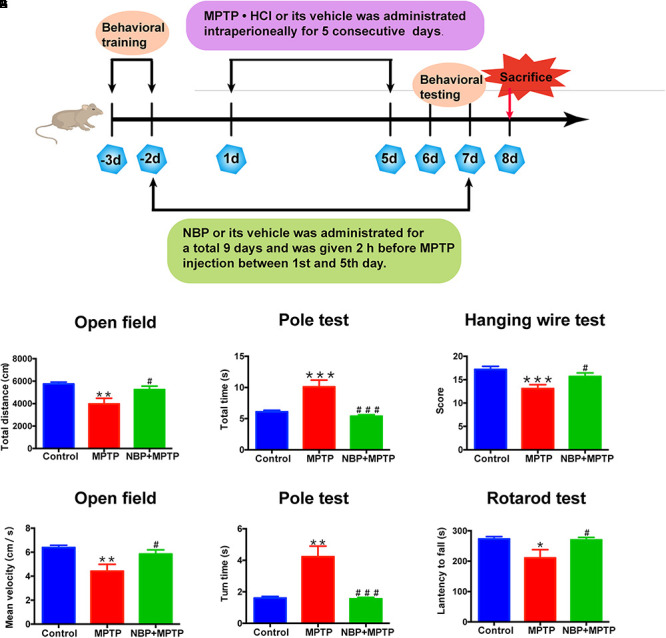
NBP improved MPTP-induced motor deficits. **(A)** The Experimental procedure and drug administration scheme. **(B,C)** Total distance **(B)** and mean velocity **(C)** of MPTP-treated mice in the open field test. **(D,E)** Total time **(D)** and turn time **(E)** spent in the pole test. **(F)** Score of hanging wire test. **(G)** Latency to fall in the rotarod test. All data are presented as means ± SEM (*n* = 10). ^∗^*p* < 0.05, ^∗∗^*p* < 0.01, ^∗∗∗^*p* < 0.001, compared with the Control group; ^#^*p* < 0.05, ^###^*p* < 0.001, compared with the MPTP group.

All animal experiments were approved by the Experimental Animal Ethics Committee of Shanghai Medical College, Fudan University and were conducted in accordance with the guidelines of the National Institutes of Health Guide for the Care and Use of Laboratory Animals. C57BL/6 male mice (10–12 weeks, 22–26 g) were purchased from Shanghai Research Center for Model Organisms (Shanghai, China) and housed at 22°C -25°C under a 12:12 h light/dark cycle. After acclimatization, the animals were randomly divided into three groups: Control, MPTP, and NBP+MPTP (*n* = 10;10;10). MPTP⋅HCl (30 mg/kg/day) was injected intraperitoneally for five consecutive days. NBP (100 mg/kg/day) was administrated intraperitoneally for 9 days and was given 2 h before injection of MPTP between the first and fifth day (Figure 1A). The dosage of NBP was chosen with reference to other brain studies of NBP in mice (Zhao et al., 2016, 2017). MPTP⋅HCl (M0896, Sigma) was dissolved in sterile normal saline, stock concentration at 5 mg/ml. NBP (HY-B0647, MCE, NJ, United States) was diluted to 10 mg/ml with vehicle (1% DMSO/2% Tween 80/45% PEG 300) immediately before use. Control group was given equal volume of solvent.

### Behavioral Testing

Mice had been trained in the pole test and rotarod test before MPTP administration (Figure 1A). Open field test, pole test, hanging wire test were carried out at first day post last MPTP injection and rotarod test was performed at second day post last MPTP injection.

Open field test: Spontaneous locomotor activity was assessed using the open field test with an automatic-recording open-field working station (MED Associates, Georgia, VT, United States). Total distance and mean velocity were analyzed over a period of 30 min.

Pole test: The pole test was used to evaluate the degree of bradykinesia and was conducted as previously described (Yu et al., 2013). This test required a vertical pole, 50 cm in height and 1 cm in diameter, with a rough surface that stood in the home cage. Mice were placed near the top of the pole, with their heads up. Time to climb down (total time) and time to turn around were recorded.

Hanging wire test: Hanging wire test was carried out to assess coordination ability. Mice were placed by forepaws at the middle of the horizontal iron wire (2 mm in diameter, 50 cm long, 35 cm high between two poles). The suspended mice tended to support themselves with their hind paws to avoid falling and to walk along the wire to reach the platform. The number of falls (up to a maximum of 10) and reaches (up to a maximum of 10) during a period of 180 s were recorded. An aggregate score from the number of falls and reaches was derived using the formula: (10-falls + reaches) [Putten et al., 2012).

Rotarod test: The rotarod test was performed on a rotarod test instrument (ENV-577M, MED Associates, United States). Mice were tested by a constant accelerating mode at 0.1 rpm/s from initial 4 rpm within a maximum recording time of 240 s. Data were collected from three trials separated by 40 min intervals. The latency to fall was calculated as the average time to fall down in three trials.

### Tissue Preparation

At third day post the last MPTP injection, mice were sacrificed and brain tissue was collected for further analysis. For protein detection, striatum was dissected rapidly on ice, frozen in liquid nitrogen and stored at -80°C. For histologic analysis, brain samples were collected and fixed in 4% paraformaldehyde at 4°C overnight. They were dehydrated sequentially in 20 and 30% sucrose solution at 4°C till sinking to the bottom of the tube. Coronal sections (30 μm) were serially cut by using a frozen microtome (Leica, Wetzlar, Germany) and stored at -20°C in anti-freeze solution.

### High-Performance Liquid Chromatography

The frozen striatum was homogenized in 0.4 M HClO_4_ and centrifuged at 12,000 rpm for 15 min at 4°C to precipitate protein. The supernatants were collected to detect monoamine concentrations. The concentrations of DA and its metabolites DOPAC and HVA, as well as 5-HT and its metabolite 5-hydroxyindoleacetic acid were determined by HPLC with electrochemical detection. Supernatant of striatum was directly added into the chromatograph (ESA, Inc., Chelmsford, MA, United States) with a 5014B electrochemical detector under analytical conditions.

### Immunohistochemical Staining

Brain sections were permeabilized with 0.6% H_2_O_2_ for 45 min to cleavage endogenous peroxidases. After washing in PBS, sections were blocked in PBS containing 0.2% Triton X-100 and 10% goat serum at 37°C for 1 h, and then incubated with primary antibody at 37°C for 2 h and overnight at 4°C. On the second day, the brain sections were transferred to secondary antibody (1:200, Vector Laboratories, CA, United States) at 37°C for 1 h and to AB peroxidase (1:200, Vector Laboratories) at 37°C for 45 min. DAB solution (Vector Laboratories) was used to visualize the staining.

### Immunofluorescence Staining

To achieve better staining result, the brain sections were treated with sodium citrate-hydrochloric acid buffer solution for antigen retrieval before staining. The brain sections or BV-2 cells with different treatments were permeabilized with 0.2% Triton X-100 in PBS at room temperature for 30 min and blocked with 10% goat serum at 37°C for 1 h, and followed by incubation with primary antibody at 37°C for 2 h and overnight at 4°C. After washing, they were incubated with secondary antibody (1:500, Invitrogen, NY, United States): donkey anti-mouse Alexa Fluor 594 or donkey anti-rabbit Alexa Fluor 488, or donkey anti-rabbit Alexa Fluor 594, or donkey anti-mouse Alexa Fluor 488. Then the brain sections were incubated with DAPI (1:1,000, Thermo Fisher, MA, United States) at room temperature for 30 min. Images were captured under a Leica confocal microscope (TCS SP-2, Leica, Wetzlar, Germany).

### Stereological Cell Counting of TH Positive Cells in the SN

Stereological cell counting was conducted to assess the density of TH positive cells in the SN as previously described (Liu et al., 2015). Briefly, a total of six sections from bregma -2.80 to -3.64 mm were collected and stained with anti-TH antibody by DAB method. The counting was performed at 40× magnification using a microscope (Olympus, Tokyo, Japan) and Stereo Investigator software (MBF Bioscience, VT, United States). The total numbers of TH positive cells were generated with the optical fractionator probe.

### Densitometric Analysis of TH Positive Fiber in the Striatum

Densitometric analysis of TH positive fibers in the striatum was performed as previously published (Liu et al., 2015). Shortly, a total of four sections from bregma +1.60 to 0.00 mm were selected and stained with anti-TH antibody by DAB method. The striatum was captured at 4× magnification with a light microscope (Leica, Wetzlar, Germany). The analysis was conducted by an IMAGE PRO PLUS system (vision 6.0, Media Cybernetics). A square 400 μm × 400 μm frame was placed in the dorsal part of the striatum to measure the density of TH positive fibers. After subtraction of background density values of the corpus callosum, the average of all sections from each animal was calculated.

### Protein Extraction and Western Blot Analysis

Total protein was exacted from cell or brain using RIPA lysis buffer (89901, Thermo, United States) with Halt Protease Inhibitor Cocktail (1861278, Thermo, United States) and Phosphatase Inhibitor Cocktail (B15001, biotool, United States). Nuclear and cytoplasmic fraction of BV-2 cells were isolated with NE-PER Nuclear and Cytoplasmic Extraction Reagents (78835, Thermo, United States) according to the manufacturer’s instructions. Protein concentrations were detected with a BCA kit (23225, Pierce, United States). The blotting membrane was blocked with 5% skim milk at room temperature for 1 h and incubated with primary antibodies for 2 h at room temperature and then at 4°C overnight. The used primary antibodies were as follows: mouse anti-actin (1:1,500 dilution, AM1021B, Abgent, United States), mouse anti-GADPH (1:1,500 dilution, 60004-1-Ig, Proteintech, United States), mouse anti-H3 (1:1,500 dilution, 17168-1-AP, Proteintech, United States), mouse anti-TH (1:1,500 dilution, T2928, sigma, United States), goat anti-IL-1β (1:1,000 dilution, AF-401-NA, Novus, CO, United States), mouse anti-TNF-α, rabbit anti-COX-2 (1:1,000 dilution, ab15191, Abcam, United Kingdom), rabbit anti-iNOS (1:1,000 dilution, ab15323, Abcam, United Kingdom), rabbit anti-p-JNK (1:1,000 dilution, 4668, CST, United States), rabbit anti-JNK (1:1,000 dilution, 9252, CST, United States), rabbit anti-p-ERK (1:1,000 dilution, A5036, Selleck, United States), rabbit anti-ERK (1:1,000 dilution, A5029, Selleck, United States), rabbit anti-p-p38 (1:1,000 dilution, CY6391, abway, China), rabbit anti-p38 (1:1,000 dilution, A5017, Selleck, United States), rabbit anti-p-p65 (1:1,000 dilution, 3033, CST, United States), mouse anti-p65 (1:1,000 dilution, Sc-8008, Santa Cruz, United States), rabbit anti-p-PI3K (1:1,000 dilution, 4228, CST, United States), rabbit anti-PI3K (1:1,000 dilution, 4257, CST, United States), rabbit anti-p-Akt (1:1,000 dilution, 4060, CST, United States), rabbit anti-Akt (1:1,000 dilution, 9272, CST, United States). After washing, secondary antibody such as IRDye 680LT goat anti-mouse IgG (H+L) (926-68020, Li-Cor, United States) or IRDye 800CW goat anti-rabbit IgG (H+L) (926-32211, Li-Cor, United States) was added for 1 h at room temperature. The proteins were finally detected using an Odyssey infrared imaging system (Li-Cor Bioscience, NE, United States). The protein levels were quantified by densitometry analysis using Quantity One 4.6.2 software (Bio-Rad, CA, United States).

### Cell Culture and Reagents

The immortalized murine microglial cell line BV-2 cells and human neuroblastoma SH-SY5Y cells were routinely maintained in Dulbecco’s Modified Eagle’s Medium with 10% fetal bovine serum, supplemented with 100 U/ml penicillin and 100 μg/ml streptomycin at 37°C in a humidified atmosphere of 95% air, and 5% CO_2_. All reagents for cell culture were purchased from Gibco BRL (Gibco, NY, United States). LPS (*Escherichia coli* serotype 055: B5, L2654) and MPP^+^ were obtained from Sigma-Aldrich (St. Louis, MO, United States).

### CM Collection and Cell Viability Assays

BV-2 cells (2 × 10^5^ cells/well in a 24-well plate) were pretreated with different concentrations of NBP for 1 h and then stimulated with LPS (0 or 100 ng/ml) or MPP^+^ (0 or 500 μM) for 24 h. The culture media were collected as CM after centrifugation at 2,000 rpm for 5 min. SH-SY5Y cells (7,500 cells/well in a 96-well plate) were seeded 1 day before the CM test. The CM from LPS or MPP^+^-treated BV-2 cells was added to SH-SY5Y cells, which were further incubated at 37°C for 24 h, and then cell viability was measured using the CCK8 assay according to the protocol.

### RNA Extraction and PCR Assay

Total RNA was isolated from cell using TRNzol Universal Reagent Kit (Tiangen, Beijing, China) mainly according to the manufacturer’s instructions. The TRNzol volume was 1,000 μl and the final elute volume was 40 μl. Single strand cDNA was synthesized using FastQuant RT Kit (with gDNase) kit (Tiangen, Beijing, China) following the conditions: 42°C for 15 min and then 95°C for 3 min. The expression of RNA was tested by real-time PCR using SYBR Green master mix (Tiangen, Beijing, China) on ABI 7300 PCR machine (Applied Biosystems, Foster City, United States). The reaction conditions were as follows: 95°C for 15 min followed by 40 cycles of 95°C for 10 s, 60°C for 20 s and 72°C for 20 s. The PCR primer sequences were as follows: mouse *actin*, 5′ CAGGATGCAGAAGGAGATTAC 3′ (forward) and 5′ AACGCAGCTCAGTAACAGTC 3′ (reverse); mouse *TNF-α*, 5′ CACGCTCTTCTGTCTACTGAACTTC 3′ (forward) and 5′ GCAGCCTTGTCCCTTGAAGAGAACC 3′ (reverse); mouse *IL-1*β, 5′ GCAACTGTTCCTGAACTC 3′ (forward) and 5′ CTCGGAGCCTGTAGTGCA 3′ (reverse); mouse *IL-6*, 5′ CATAGCTACCTGGAGTACATGA 3′ (forward) and 5′ CATTCATATTGTCAGTTCTTCG 3′ (reverse); mouse *COX-2*, 5′ GTTCATCCCTGACCCCCAAG 3′ (forward) and 5′ ACTCTGTTGTGCTCCCGAAG 3′ (reverse); mouse *iNOS*, 5′ CCCTTCCGAAGTTTCTGGCAGCAGC 3′ (forward) and 5′ GGCTGTCAGAGCCTCGTGGCTTTGG 3′ (reverse). The relative expression of interested gene was normalized to the endogenous control *actin* expression using the comparative cycle threshold method. When comparing different groups, the data were logarithm-transformed.

### Measurement of Cytokines, NO and Intracellular ROS

BV-2 cells (2 × 10^5^ cells per well in a 24-well plate or 1 × 10^4^ cells per well in a 96-well plate) were pretreated with NBP (0 or 100 μM) for 1 h and further stimulated with LPS (0 or 100 ng/ml) for 24 h. Culture medium was collected and cellular debris was eliminated through centrifugation at 2,000 rpm for 5 min. Cytokines in supernatants were assayed by ELISA kits. Concentrations of IL-1β was measured by using procedures recommended by the supplier (R&D, MN, United States) and TNF-α was detected according to the protocol (ABclonal, Wuhan, China).

Accumulated nitrite in supernatants were measured using Griess reagent (Beyotime, Shanghai, Beijing). Briefly, 50 μl of supernatant were transferred to 96-well plates and absorbance was measured at 540 nm on a microplate reader (Epoch, BioTek, VT, United States) after mixture with Griess regents. The nitrite concentration was calculated with reference to the standard curve generated with NaNO_2_.

Intracellular accumulation of ROS were measured by DCFH-DA assay (Beyotime, Shanghai, Beijing). After drug treatment, BV-2 cells were incubated with 40 μM DCFH-DA at 37°C for 45 min. After washing three times with PBS, the intensity of fluorescence was read at 488 nm excitation and 530 nm emission on a fluorescence plate reader (Fluoroskan Ascent, Thermo Fisher Scientific, MA, United States). The obtained values were presented as folds of the controls. The images of DCFH-DA in BV-2 cells were captured under a microscope (Nikon Eclipse Ti-s, Nikon, Tokyo, Japan).

### Statistical Analysis

Data were analyzed using Prism 6.0 software (GraphPad Software, San Diego, CA, United States). All values are expressed as the means ± SEM and analyzed by one-way ANOVA followed by multiple comparisons with the least significant difference (LSD) *post hoc* test. Differences are considered significant at *p* < 0.05.

## Results

### NBP Improved MPTP-Induced Motor Deficits

MPTP is a commonly used neurotoxin in PD researches, which selectively damages dopaminergic neurons and induces PD like behavior in animals (Jagmag et al., 2015). To explore the neuroprotective effect of NBP in PD, we established a sub-acute MPTP model of PD in adult male mice and tested whether NBP improved MPTP-induced motor deficits. The delivery of NBP into brain was proven by HPLC as Supplementary Figure S1 showed. Behavioral tests including open field, pole test, hanging wire test and rotarod test were performed. Open field is a well-established and widely used paradigm for evaluating general motor behavior in PD animals (Asakawa et al., 2016). As shown in Figure 1B,C, the MPTP group displayed a significant reduction in total movement distance and mean velocity compared to the Control group. Administration of NBP significantly rescued these changes. We then performed the pole test to check whether PD mice exhibited the feature of bradykinesia. MPTP injection remarkably prolonged the time for mice to climb down as well as turning around (Figure 1D,E). However, the NBP+MPTP mice displayed a better performance in the pole test compared with those in MPTP group. Hanging wire test and rotarod test were further carried out to evaluate coordination ability in PD mice. As shown in Figure 1F,G, MPTP treatment decreased the score in the hanging wire test and shortened the latency to fall in the rotarod test. Treatment with NBP significantly improved the performance in both hanging wire test and rotarod test compared with the MPTP group. Collectively, these results suggest that NBP administration improved MPTP-induced motor impairment.

### NBP Ameliorated MPTP-Induced Nigrostriatal Dopaminergic Injury

**Figure 2:**
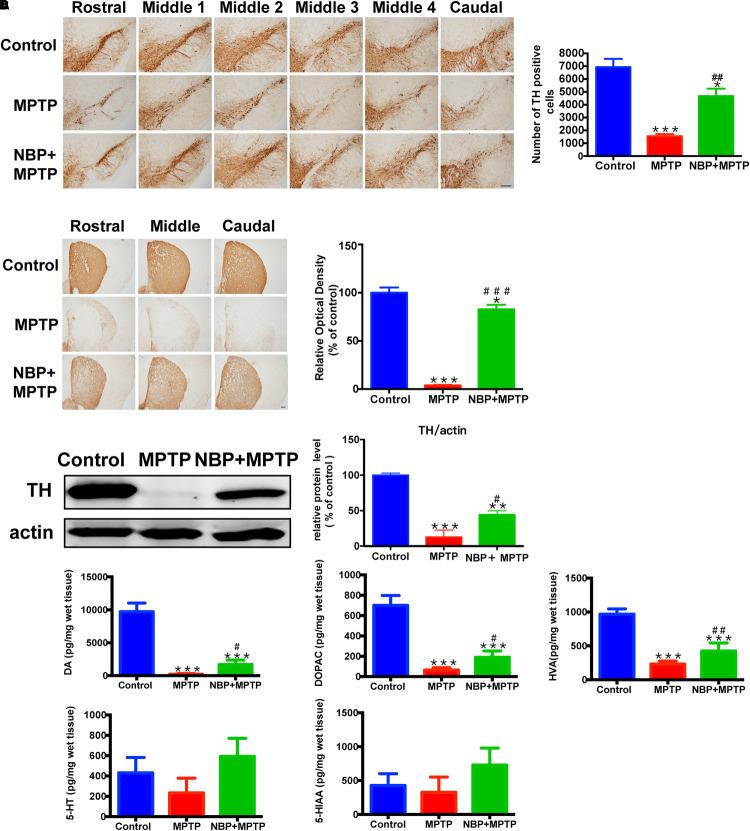
NBP ameliorated MPTP-induced nigrostriatal dopaminergic injury. **(A)** Immunohistochemical staining showing TH positive cells in the SN (scale bar: 0.2 mm). Stereological counting of TH positive cells is shown in the right panel (*n* = 4). **(B)** Immunohistochemical staining showing striatal TH positive nerve fibers (scale bar: 0.2 mm). Densitometric analysis of the relative optical density of TH staining is shown in the right panel (*n* = 4). **(C)** Western Blot analysis of striatal TH protein (*n* = 4). Protein band intensity is normalized to actin and is expressed as fold difference relative to the Control group. **(D)** HPLC assays of DA, DOPAC, HVA, 5-HT and 5-HIAA (*n* = 5). All data are presented as means ± SEM. ^∗^*p* < 0.05, ^∗∗^*p* < 0.01, ^∗∗∗^*p* < 0.001, compared with the Control group; ^#^*p* < 0.05, ^##^*p* < 0.01, ^###^*p* < 0.001, compared with the MPTP group.

The typical pathological characteristics of PD include the loss of dopaminergic neurons in the SN and dopaminergic terminals in the striatum. To determine whether NBP could protect dopaminergic neurons from MPTP damage, we detected nigral TH positive cells by immunohistochemistry and performed stereological counting. As is shown in Figure 2A, the number of TH positive cells in the SN was dramatically reduced by MPTP injection, only 22.4% of TH positive cells survived from the neurotoxicity. Pretreatment with NBP reversed MPTP-induced loss of dopaminergic neurons in the SN (67.5% of the control). The effect of MPTP and NBP on striatal dopaminergic terminals was also examined by immunohistochemistry staining and Western Blot. Densitometric analysis showed that MPTP treatment caused a prominent depletion of TH positive nerve fibers in the striatum, only 3.9% of striatal TH positive nerve fibers remained intact (Figure 2B). The expression of TH protein was reduced by MPTP to 4.3% of the control group as shown by Western Blot assay (Figure 2C). Treatment of NBP significantly rescued the density of striatal TH positive nerve fibers (82.8% of the control) and TH protein level (37.8% of the control) (Figure 2B,C). Injection of NBP alone did not affect the nigrostriatal axis, including the striatal TH protein level and the density of striatal TH positive nerve fibers and the number of TH positive neurons in the SN (Supplementary Figure S2).

The abundance of DA, and its metabolites DOPAC and HVA, also 5-HT and its metabolite 5-HIAA in the striatum were measured by HPLC. As is shown in Figure 2D, MPTP treatment resulted in reduction in the striatal levels of DA, DOPAC and HVA. However, administration of NBP significantly rescued MPTP-induced depletion of DA, DOPAC and HVA (Figure 2D). The amount of 5-HT and 5-HIAA were not affected by MPTP or NBP treatment. These results indicated that NBP treatment blocked MPTP-induced loss of DA and its metabolites in the striatum. Overall, NBP ameliorated MPTP-induced nigrostriatal dopaminergic injury.

### NBP Suppressed MPTP-Induced Activation of Microglia in the Striatum and SN

**Figure 3:**
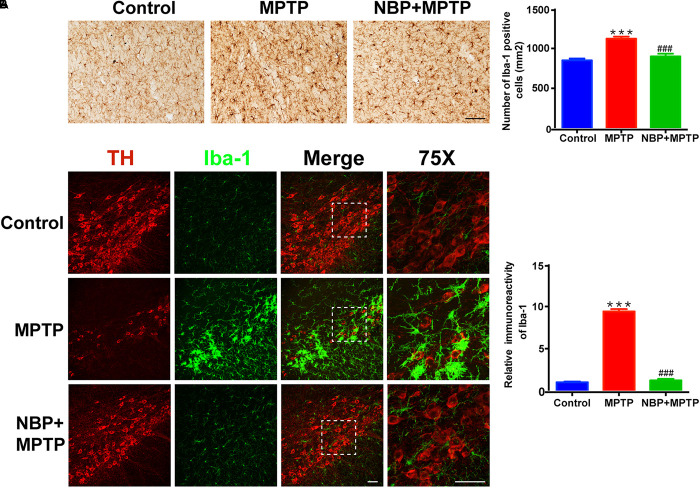
NBP suppressed MPTP-induced activation of microglia in the striatum and SN. **(A)** Immunohistochemical staining showing Iba-1 positive cells in the striatum (scale bar: 0.1 mm). Counting of Iba-1 positive cells in the striatum is shown in the right panel (*n* = 4). **(B)** Double immunofluorescence staining of TH (red) and Iba-1 (green) in the SN (scale bar: 50 μm). Relative immunoreactivity intensity of Iba-1 is shown in the right panel (*n* = 3). All data are presented as means ± SEM. ^∗∗∗^*p* < 0.001, compared with the Control group; ^###^*p* < 0.001, compared with the MPTP group.

In line with previous studies, we also observed robust microglial activation in the striatum and SN in MPTP-intoxicated mice (Hou et al., 2017; Lecca et al., 2018). The activation of microglia was investigated by using immunohistochemical staining and immunofluorescence staining for Iba-1 positive cells. The numbers of Iba-1 positive cells in the striatum and SN both greatly increased in MPTP group (Figure 3A,B). Moreover, the morphological changes of microglia such as larger cell bodies and more branches were obvious especially in the SN after MPTP treatment. NBP treatment significantly inhibited MPTP-induced microglial activation in the striatum and SN. As to MPTP-induced activation of astrocyte, we did not find any difference between MPTP group and NBP+MPTP group in the striatum and SN (Supplementary Figure S3).

### NBP Reduced the Expression of Inflammatory Mediators and Inhibited the MAPK-ERK Pathway in MPTP-Treated Mice

**Figure 4:**
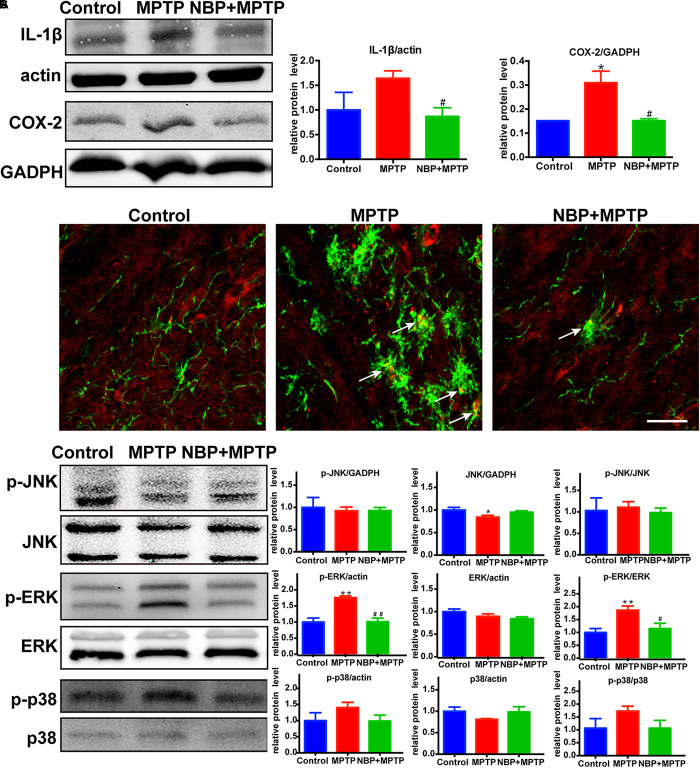
NBP reduced the expression of inflammatory mediators and inhibited the MAPK-ERK pathway in MPTP-treated mice. **(A)** Western Blot analysis of striatal IL-1β and COX-2 protein. **(B)** Double immunofluorescence staining of iNOS (red) and Iba-1 (green) in the SN (scale bar: 25 μm). **(C)** Western Blot assay for MAPKs pathway. All data are presented as means ± SEM (*n* = 3–4). ^∗^*p* < 0.05, ^∗∗^*p* < 0.01, compared with the Control group; ^#^*p* < 0.05, ^##^*p* < 0.01, compared with the MPTP group.

It is widely accepted that inflammatory mediators derived from microglia aggravate the progression of neuronal cell deaths in PD (Glass et al., 2010; Ransohoff, 2016). MPTP-treated mice showed higher expression of IL-1β and COX-2 in the striatum (Figure 4A). MPTP-induced upregulation of these inflammatory mediators were reversed by NBP treatment. Since the level of striatal iNOS was too low for detection by Western Blot in our study, we analyzed the expression of iNOS in the SN by immunofluorescence staining (Figure 4B). In the Control group, we hardly found iNOS expression in Iba-1 positive cells, while in the MPTP group the number of double staining cells increased. Furthermore, treatment of NBP reduced the expression of iNOS in nigral microglia.

The MAPK pathways which played an important role in microglial activation were reported to be regulated by NBP in several neurological diseases (Kaminska et al., 2009; Lu et al., 2012; Bhatia et al., 2016; Jiang et al., 2017; Yan R. Y. et al., 2017). Thus we speculated that the protective role of NBP might be associated with the regulation of MAPK pathways. The signaling pathway of ERK but not JNK nor p38, was activated after MPTP injection in our study (Figure 4C). Moreover, MPTP-induced phosphorylation of ERK was significantly reduced in NBP+MPTP mice compared to the MPTP group.

### NBP Protected Dopaminergic Neurons From Neurotoxicity Induced by Microglial Activation

**Figure 5:**
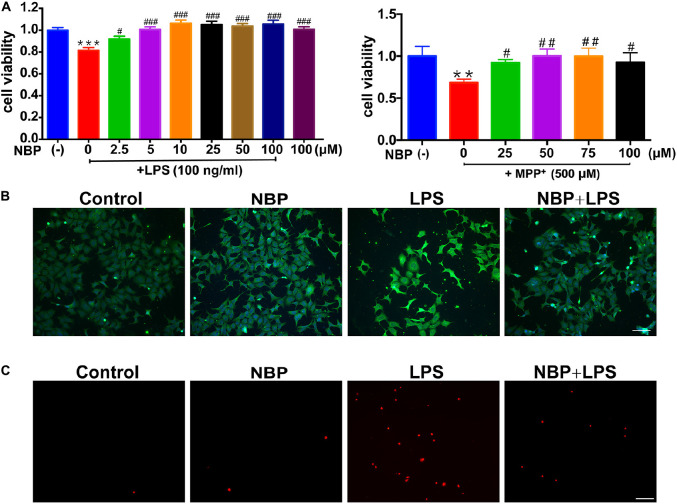
NBP protected dopaminergic neurons from neurotoxicity induced by microglial activation. SH-SY5Y cells were incubated for 24 h with conditioned medium derived from cultures of BV-2 cells. Before collecting culture media, BV-2 cells were pretreated with NBP (0 or 100 μM) for 1 h and incubated with LPS (0 or 100 ng/ml) or MPP^+^ (0 or 500 μM) for 24 h. **(A)** Cell viability was measured with the CCK8 assay (*n* = 5). All data are presented as means ± SEM. ^∗∗^*p* < 0.01, ^∗∗∗^*p* < 0.001, compared with the Control group; ^#^*p* < 0.05, ^##^*p* < 0.01, ^###^*p* < 0.001, compared with the LPS group or the MPP^+^ group. **(B)** The apoptosis of SH-SY5Y was evaluated by immunofluorescence detection of cleaved caspase-3 (green) and cell nuclei was stained with DAPI (blue) (scale bar: 50 μm). **(C)** The cell death of SH-SY5Y was evaluated by immunofluorescence detection of PI (red) (scale bar: 50 μm).

Since the activation of microglia but not astrocyte was reversed by NBP treatment *in vivo* (Figure 3 and Supplementary Figure S3), we further investigated the anti-inflammatory activity of NBP in BV-2 microglia treated with LPS or MPP^+^. We first conducted cell viability assay to examine the cytotoxicity of NBP. The CCK8 assay showed that NBP did not affect cell viability within 200 μM and NBP may have cytotoxicity above 200 μM concentration (Supplementary Figure S4). When CM from LPS treated-BV-2 cells were added to SH-SY5Y cells, the viability of SH-SY5Y cells decreased by about 20% after 24 h incubation, suggesting the neurotoxicity of microglia-mediated inflammation on dopaminergic neurons (Figure 5A). CM derived from BV-2 cells with NBP administration 1 h prior to LPS treatment, however, reduced SH-SY5Y cells death in a concentration-dependent manner (2.5–100 μM). Combined with the concentration-dependent effect of NBP on pro-inflammatory cytokine mRNA expression (data not shown), 100 μM was chosen for subsequent experiments. NBP also protected SH-SY5Y cells from CM which derived from MPP^+^ treated-BV-2 cells (Figure 5A).

The cleaved caspase-3 and PI immunofluorescence staining in SH-SY5Y demonstrated NBP alleviated dopaminergic cells damage induced by activated microglia-CM at both apoptosis and cell death level. These results suggested that NBP exerted neuroprotective role at least partly by blocking microglial activation and subsequent inflammation-mediated neurotoxicity (Figure 5B,C).

### NBP Reduced Pro-inflammatory Molecules Expression in LPS-Stimulated BV-2 Cells

**Figure 6:**
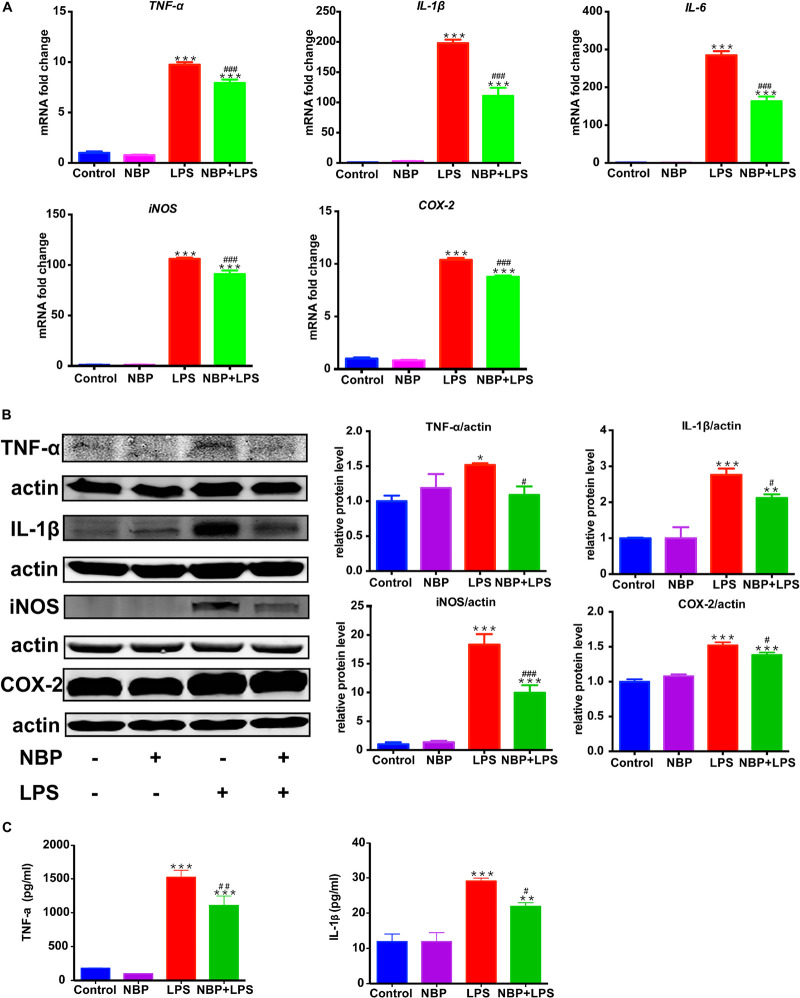
NBP reduced pro-inflammatory molecules expression in LPS-stimulated BV-2 cells. BV-2 cells were pretreated with NBP (0 or 100 μM) for 1 h followed by LPS (0 or 100 ng/ml) for 6 h and total RNA was isolated. To measure cellular protein expression or cytokines level in supernatants, time for LPS treatment was 24 h. **(A)** The mRNA expression of *IL-1*β, *IL-6, TNF-a, iNOS* and *COX-2* was analyzed by RT-PCR and normalized to that of *β-actin*. **(B)** The protein level of TNF-α, IL-1β, iNOS and COX2 was analyzed by Western Blot. **(C)** The level of TNF-α and IL-1β in supernatants was assayed by ELISA kit. All data are presented as means ± SEM (*n* = 3). ^∗^*p* < 0.05, ^∗∗^*p* < 0.01, ^∗∗∗^*p* < 0.001, compared with the Control group; ^#^*p* < 0.05, ^##^*p* < 0.01, ^###^*p* < 0.001, compared with the LPS group.

RT-PCR analysis showed that the mRNA expression of *IL-1*β, *IL-6, TNF-α, iNOS* and *COX-2* increased dramatically after LPS stimulation in BV-2 cells (Figure 6A). The upregulation of these pro-inflammatory cytokines induced by LPS was significantly attenuated by pretreatment of NBP. This data suggested that NBP regulated the expressions of pro-inflammatory cytokines at the transcriptional level. Western Blot assay further confirmed the inhibitory effect of NBP on the production of pro-inflammatory cytokines at protein level (Figure 6B). The secretion of *IL-1*β and *TNF-α* in the supernatants were also reduced significantly by NBP treatment as the ELISA assay showed (Figure 6C).

### NBP Suppressed NO Release and ROS Generation in LPS-Stimulated BV-2 Cells

**Figure 7:**
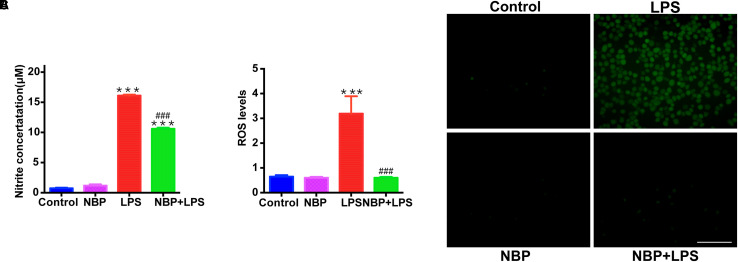
NBP suppressed NO release and ROS generation in LPS-stimulated BV-2 cells. BV-2 cells were pretreated with NBP (0 or 100 μM) for 1 h and followed by LPS (0 or 100 ng/ml) for 24 h. **(A)** NO levels in culture supernatant were measured by Griess method (*n* = 3). **(B)** ROS production in BV-2 was measured using fluorescence probe DCFH-DA and the fluorescence intensity was determined at 485 nm excitation and 535 nm emission (*n* = 5). **(C)** Immunofluorescence detection of DCFH-DA (green) (scale bar: 0.1 mm). All data are presented as means ± SEM (*n* = 3). ^∗∗∗^*p* < 0.001, compared with the Control group; ^###^*p* < 0.001, compared with the LPS group.

Besides the pro-inflammatory cytokines mentioned above, NO also serves as a vital inflammatory mediator in LPS-stimulated BV-2 microglia. We examined the effect of NBP on NO release by Griess method. NBP alone did not affect the release of NO in BV-2 cells (Figure 7A). LPS stimulation caused a robust increase of NO while NBP pretreatment blocked the NO release in activated BV-2 cells. We also investigated the ROS production which acts as a second messenger in inflammatory response. Pretreatment with NBP remarkably reduced LPS-induced ROS generation as shown by the fluorescence detection of DCFH-DA in BV-2 microglia (Figure 7B,C).

### NBP Inhibited the Activation of ERK, NF-κB and PI3K/Akt Pathways in LPS-Stimulated BV-2 Cells

**Figure 8:**
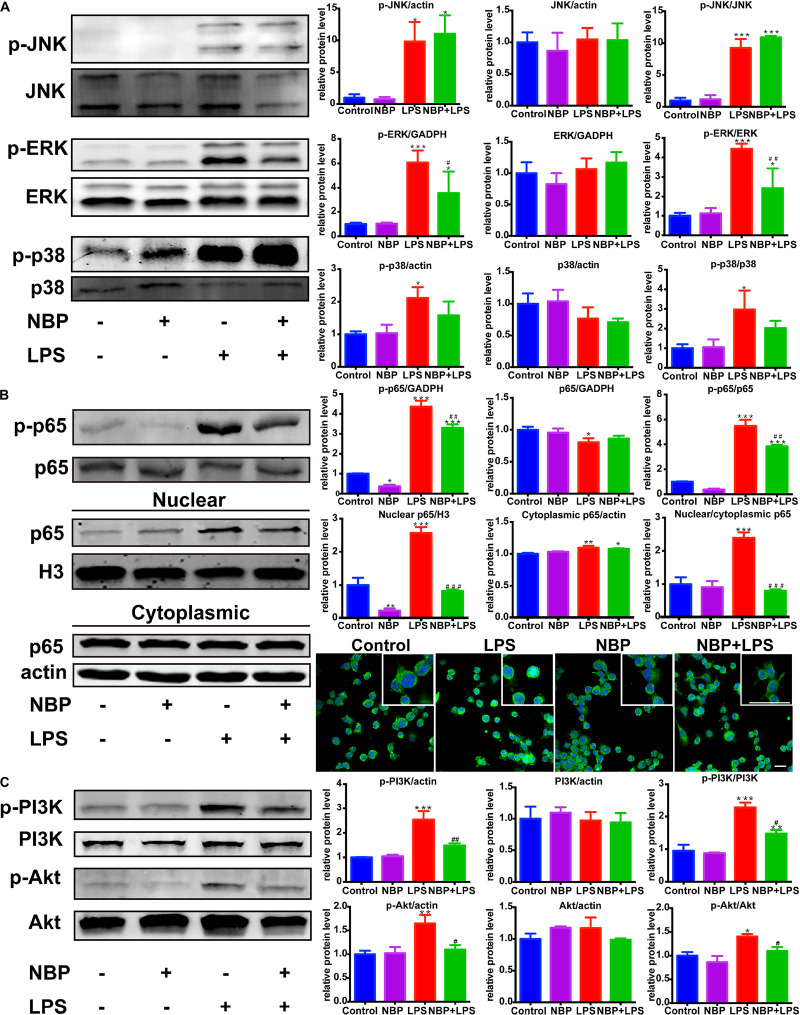
NBP inhibited the activation of ERK, NF-κB and PI3K/Akt pathways in LPS-stimulated BV-2 cells. **(A)** Western Blot assay for MAPK expression. The expression of total JNK/p38/ERK as well as p-JNK/p-p38/p-ERK were analyzed. **(B)** Western Blot assay for NF-κB expression in whole-cell, nuclear and cytoplasmic extracts. The nuclear translocation of p65 was also evaluated by immunofluorescence detection of p65 (green) and cell nuclei was stained with DAPI (blue) (scale bar: 50 μm). **(C)** Western Blot assay for PI3K/Akt expression. The expression of total PI3K/Akt as well as p-PI3K/p-Akt were analyzed. All data are presented as means ± SEM (*n* = 3). ^∗^*p* < 0.05, ^∗∗^*p* < 0.01, ^∗∗∗^*p* < 0.001, compared with the Control group; ^#^*p* < 0.05, ^##^*p* < 0.01, ^###^*p* < 0.001, compared with the LPS group.

We next investigated the effect of NBP on the MAPKs signaling in LPS-activated BV-2 microglia. Consistent with *in vivo* study, NBP had significant inhibitory effect on LPS-stimulated ERK activation while the phosphorylation of JNK and p38 MAPK were not affected (Figure 8A).

Nuclear factor kappa-light-chain-enhancer of activated B cells is considered as an essential mediator of inflammatory response and its activation is involved in the early stage of neurodegenerative diseases (Srinivasan and Lahiri, 2015). The above data showed that NBP inhibited the expression of *TNF-α*, *IL-1*β and *iNOS*, which are target genes of NF-κB. Therefore, we further explored the role of NBP in the regulation of NF-κB pathway. The activation of NF-κB p65 subunit by phosphorylation is recognized to trigger p65 nuclear translocation and induce the transcription of pro-inflammatory genes. We found that LPS-induced phosphorylation of p65 subunit was largely reduced by NBP pretreatment (Figure 8B). Moreover, Western Blot analysis of nuclear and cytoplasmic fractions showed that NBP remarkably suppressed the expression of nuclear p65 after LPS stimulation. Analysis of p65 subcellular localization by immunofluorescence assay also demonstrated that the accumulation of nuclear p65 in response to LPS stimuli can be prevented by NBP administration (Figure 8B).

Due to the reported modulation role of PI3K/Akt on NF-κB pathway, we examined the effect of NBP on PI3K/Akt signaling (Kane et al., 1999). As shown in Figure 8C, LPS treatment strongly activated PI3K/Akt signaling by upregulating the phosphorylation level. However, the increased phosphorylation of PI3K and Akt were sharply reduced by NBP in BV-2 cells.

### NBP Reduced Pro-inflammatory Molecule Expression and Inhibited the Activation of JNK and NF-κB Pathways in MPP^+^-Stimulated BV-2 Cells

**Figure 9:**
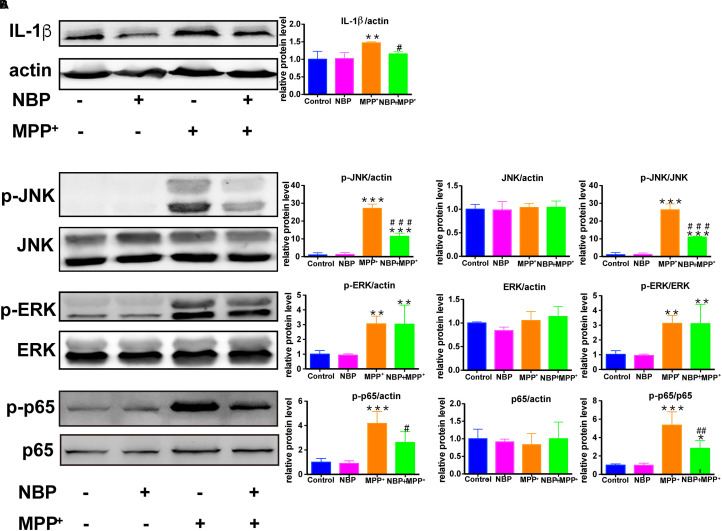
NBP reduced IL-1β level and inhibited the activation of JNK and NF-κB pathways in MPP^+^-stimulated BV-2 cells. BV-2 cells were pretreated with NBP (0 or 100 μM) for 1 h and followed by MPP^+^ (0 or 500 μM) for 24 h. **(A)** The protein level of IL-1β was analyzed by Western Blot. **(B)** Western Blot assay for MAPK and NF-κB expression. All data are presented as means ± SEM (*n* = 3). ^∗∗^*p* < 0.01, ^∗∗∗^*p* < 0.001, compared with the Control group; ^#^*p* < 0.05, ^##^*p* < 0.01, ^###^*p* < 0.001, compared with the MPP^+^ group.

We also examined the role of NBP in MPP^+^-treated BV-2 cells. MPP^+^ (500 μM) was selected to activate the pro-inflammatory phenotype of BV-2 cells without inducing severe cell death according to previous study (Jin et al., 2012) as well as our concentration experiments (Supplementary Figures S5A–C). As shown in Supplementary Figure S5B, the expression of IL-1β was increased by MPP^+^ (500 μM) in BV-2 cells while COX-2 and iNOS remained unchanged. The up-regulation of IL-1β by MPP^+^ was significantly attenuated by the pretreatment of NBP (Figure 9A). We further investigated the effect of NBP on signaling pathways. MPP^+^ treatment was found to activate JNK and ERK MAPK (Figure 9B) but not p38 (data not shown) in BV-2 cells. Unlike in LPS induced cell model, NBP inhibited MPP^+^-stimulated JNK activation but the phosphorylation of ERK was not affected. MPP^+^-induced phosphorylation of p65 subunit was also largely reduced by NBP pretreatment. PI3K/Akt pathway were not significantly activated after MPP^+^ treatment (data not shown). These results indicated that NBP also exhibited anti-inflammatory activity in MPP^+^-treated BV-2 cells.

## Discussion

The present study demonstrated that NBP exerted significant dopaminergic neuroprotection against MPTP-induced dopaminergic neurotoxicity and suppressed microglial activation. NBP significantly reduced the number of reactive microglia and attenuated MPTP-induced nigrostriatal dopaminergic degeneration and subsequent motor deficit in PD mice. *In vitro* experiments showed that NBP alleviated dopaminergic neuronal damage induced by activated microglia-CM and blocked the production of pro-inflammatory molecules as well as ROS. Furthermore, we found that the anti-inflammatory effect of NBP was possibly mediated via the suppression of MAPK, NF-κB and PI3K/Akt pathways.

In recent decades, the importance of neuroinflammation in PD progression has been realized (Ransohoff, 2016; Janda et al., 2018; Sommer et al., 2018). Microglia, a brain resident immune cell, plays a central role in the process of neuroinflammation. The activated microglia promotes the production of pro-inflammatory molecules and ROS which make dopaminergic neurons more vulnerable to degeneration or toxicity (Ransohoff, 2016). Besides, microglia-mediated neuroinflammation accelerates neuronal accumulation of α-synuclein which is a typical pathological feature of PD (Sanchez-Guajardo et al., 2015). Therefore, inhibition of microglial activation and neuroinflammation is considered as a promising strategy for treating PD. In the present study, we observed that the number of activated microglia with larger cell bodies and shorter processes decreased remarkably in the striatum and SN after NBP administration. The activation of astrocyte stimulated by MPTP was barely affected by NBP, suggesting a selective role of NBP in glia cells. NBP significantly reduced the levels of inflammatory mediators including IL-1β, iNOS and COX-2 *in vivo* and *in vitro*. Our results, for the first time, demonstrated the anti-inflammatory activity of NBP in MPTP-induced mouse model of PD. Another group recently reported the anti-inflammatory activity of NBP in LPS-stimulated PD model of mice but further molecular mechanism was not demonstrated (Chen et al., 2018).

Mitogen-activated protein kinase signaling pathways play vital roles in neuroinflammation triggered by LPS and MPTP (Bhatia et al., 2016; Ren et al., 2018). NBP was found to regulate JNK and p38 MAPK pathway in a cell model of AD and a rat model of stroke (Lei et al., 2014; Yan R. Y. et al., 2017). In our study, the activation of ERK signaling in the striatum of MPTP-intoxicated mice was largely reduced by NBP treatment. *In vitro* experiment of LPS-treated BV-2 cells showed that NBP strongly inhibited LPS-induced phosphorylation of ERK whereas did not affect the phosphorylation of JNK and p38. While in MPP^+^-stimulated BV-2 cells, it was not ERK but JNK pathway be suppressed by NBP. The activation of ERK and JNK were reported to enhance the transcriptional activity of AP-1 which promotes the expression of many pro-inflammatory genes such as *TNF-α*, *IL-1*β, *IL-6* (Johnson and Lapadat, 2002; Ye et al., 2014).

NF-κB, a transcription factor of more than 500 target genes, serves as a key regulator of inflammatory response (Gupta et al., 2010). Specific binding regions of NF-κB have been identified in pro-inflammatory genes including *TNF-α*, *IL-1*β, *IL-6*, *iNOS* and *COX-2*. Increasing evidence for an involvement of NF-κB in PD progression has accumulated in recent years (Tiwari and Pal, 2017). NF-κB pathway was reported to be activated within the SN pars compact of PD patients and experimental animals (Hunot et al., 1997; Cao et al., 2008). Selective inhibition of NF-κB activation was shown to alleviate dopaminergic neuronal loss (Ghosh et al., 2007). In our study, NBP administration significantly suppressed LPS-mediated as well as MPP^+^-mediated nuclear translocation of NF-κB and reduced the production of the above-mentioned pro-inflammatory genes, suggesting that inactivation of NF-κB at least partly contribute to the anti-inflammatory effect of NBP in BV-2 microglia. The effect of NBP on NF-κB pathway in sub-acute MPTP induced PD mouse model needs further research.

**Figure 10:**
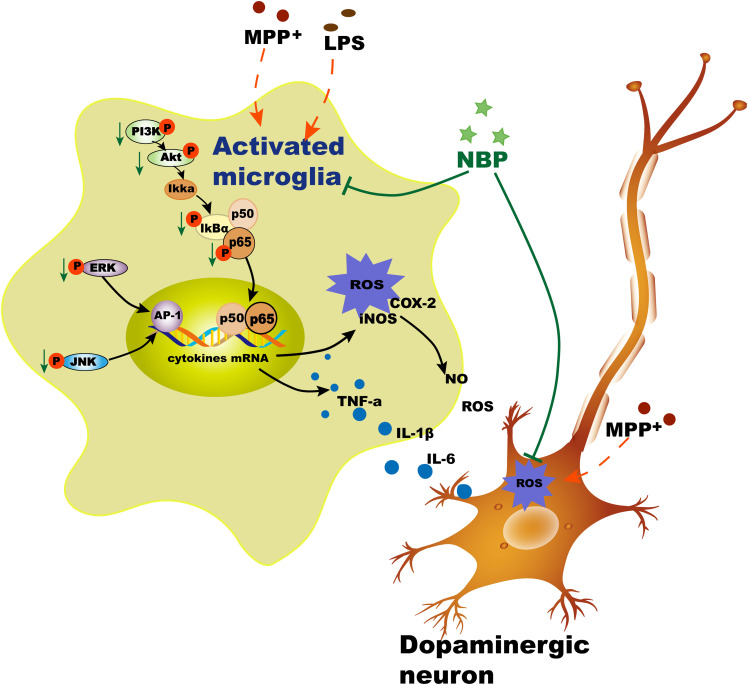
Schematic illustration demonstrates that NBP protects dopaminergic neurons from inflammation via inhibition of MAPK, NF-κB and PI3K/Akt pathways in microglia.

We also examined the effects of NBP on PI3K/Akt pathway in LPS-stimulated microglia. PI3K/Akt pathway had been reported to be involved in COX-2 and iNOS production in response to various stimuli including LPS (Vyas and Vohora, 2017). Moreover, Akt could also activate NF-κB pathway via regulating IκB degradation by phosphorylation of IKK in activated BV-2 cell (Kane et al., 1999). Our result showed that NBP significantly inhibited the phosphorylation of PI3K and Akt in LPS-stimulated BV-2 cells. However, the dual roles of PI3k/Akt pathway in triggering inflammation were inhibited by the treatment of NBP. Collectively, NBP produced anti-inflammatory effect via inhibition of MAPK, NF-κB and PI3K/Akt pathways (Figure 10). So far the exact mechanism of NBP-mediated protection is not yet understood and whether there is a unique upstream target that control these pathways requires more exploration.

The dilemma of turning neuroprotective drugs into PD therapy are the difficulties in delivery and side effects. Drug repurposing strategy has drawn great interest because of the vastly saved investment in time and money. Since NBP has been widely used clinically in treating patients with ischemic stroke for years, the efficacy of crossing blood–brain barrier as well as safety issue have been well settled. Moreover, previous studies had indicated that NBP may have a multi-targeted neuroprotective effect such as mitochondrial protection, anti-oxidative stress, anti-apoptotic and so on. Our research primarily explored the anti-inflammatory activity and mechanism of NBP in PD models. Most likely, NBP may arrest PD progression via a combination effect. It is delightful to see that there are two ongoing clinical trials (ChiCTR1800018892; ChiCTR-IPR-16009395) which take NBP to treat PD patients.

In conclusion, we demonstrated that NBP entered into the brain, inhibited the expression of pro-inflammatory molecules and microglial activation, alleviated nigrostriatal dopaminergic degeneration, and improved motor functions in MPTP-intoxicated mice. The anti-neuroinflammatory mechanism may be closely associated with the inhibition of MAPK, NF-κB and PI3K/Akt pathways. Our results suggested that NBP might have potential for therapeutic intervention in PD and other neurodegenerative disorders where inflammation plays an important role in disease pathogenesis.

## Data Availability

All datasets generated for this study are included in the manuscript and/or the Supplementary Files.

## Ethics Statement

This study was carried out in accordance with the recommendations of the guidelines of the National Institutes of Health Guide for the Care and Use of Laboratory Animals. The protocol was approved by the Experimental Animal Ethics Committee of Shanghai Medical College, Fudan University.

## Author Contributions

YC designed the study, performed the experiments, and wrote the manuscript. MY, FH, and Y-CW supervised the design of the study, analyzed the data, and refined the manuscript. S-HK revised the manuscript. TW, XL, and XZ contributed to the animal behavior tests and the immunoassays. HL and QL participated in the cell studies. All authors mentioned in the paper have significantly contributed to the research. All authors read and approved the final manuscript.

## Conflict of Interest Statement

The authors declare that the research was conducted in the absence of any commercial or financial relationships that could be construed as a potential conflict of interest.

## Abbreviations

5-HIAA: 5-hydroxyindoleacetic acid
5-HT: serotonin
AP-1: activator protein-1
CM: conditioned medium
COX: cyclooxygenase
DA: dopamine
DOPAC: 3,4 dihydroxyphenylacetic acid
ERK: extracellular signal-regulated kinase
HPLC: high-performance liquid chromatography
HVA: homovanillic acid
IL: Interleukin
iNOS: inducible nitric oxide synthase
LPS: lipopolysaccharide
MAPK: mitogen-activated protein kinase
MPTP: 1-methyl-4-phenyl-1,2,3,6-tetrahydropyridine
NBP: Dl-3-*n*-butylphthalide
NF-κB: nuclear factor kappa-light-chain-enhancer of activated B cells
NO: nitric oxide
PBS: phosphate buffer saline
PD: Parkinson’s disease
ROS: reactive oxygen species
SEM: standard error of mean
SN: substantia nigra
TH: tyrosine hydroxylase
TNF: tumor necrosis factor.
