# Corrigendum: Dl-3-*n*-Butylphthalide Exerts Dopaminergic Neuroprotection Through Inhibition of Neuroinflammation

**DOI:** 10.3389/fnagi.2021.620015

**Published:** 2021-07-06

**Authors:** Yajing Chen, Tingting Wu, Heng Li, Xuan Li, Qing Li, Xiaoying Zhu, Mei Yu, Sheng-Han Kuo, Fang Huang, Yun-Cheng Wu

**Affiliations:** ^1^Department of Neurology, Shanghai General Hospital, Shanghai Jiao Tong University School of Medicine, Shanghai, China; ^2^Department of Neurology, Jinan Central Hospital Affiliated to Shandong University, Jinan, China; ^3^The State Key Laboratory of Medical Neurobiology, The Institutes of Brain Science and the Collaborative Innovation Center for Brain Science, Shanghai Medical College, Fudan University, Shanghai, China; ^4^Department of Neurology, College of Physicians and Surgeons, Columbia University, New York, NY, United States

**Keywords:** dl-3-*n*-butylphthalide, MAPK, microglia, neuroinflammation, NF-κB, Parkinson's disease

In the original article, there was a mistake in Figure 5B, Figure 6B, and Figure 8B as published. The image in Figure 5B (NBP) was inadvertently replaced with the image from Figure 5B (NBP+LPS). In Figure 6B, the extra bands of COX-2 were mistaken as the main bands. In Figure 8B, the images of cytoplasmic p65 and actin bands were inadvertently exchanged during images organization. The corrected Figure 5, Figure 6, and Figure 8 appear below.

**Figure 5:**
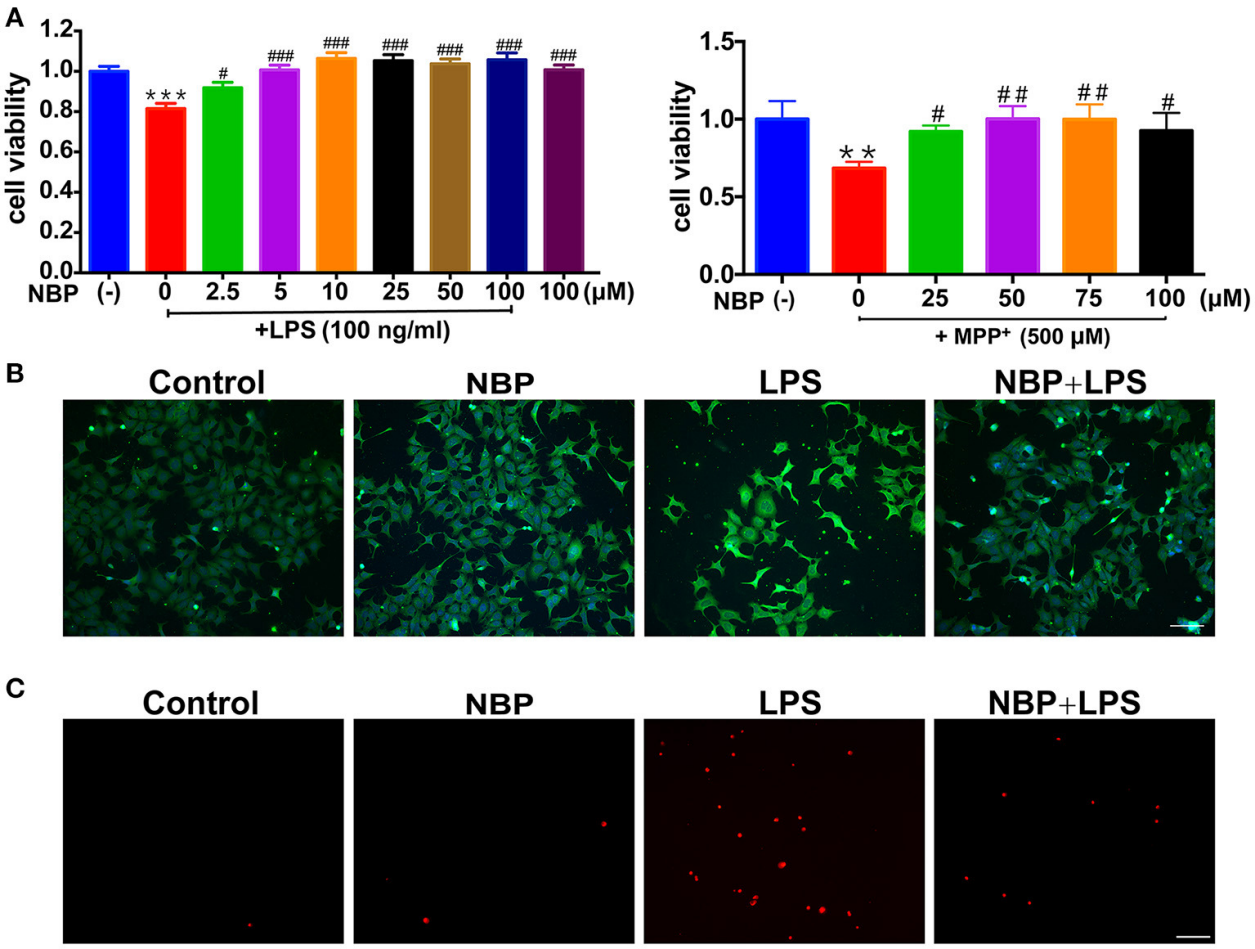
NBP protected dopaminergic neurons from neurotoxicity induced by microglial activation. SH-SY5Y cells were incubated for 24 h with conditioned medium derived from cultures of BV-2 cells. Before collecting culture media, BV-2 cells were pretreated with NBP (0 or 100 μM) for 1 h and incubated with LPS (0 or 100 ng/ml) or MPP^+^ (0 or 500 μM) for 24 h. **(A)** Cell viability was measured with the CCK8 assay (*n* = 5). All data are presented as means ± SEM. **p < 0.01, ***p < 0.001, compared with the Control group; ^#^*p* < 0.05, ^##^*p* < 0.01, ^###^*p* < 0.001, compared with the LPS group or the MPP^+^ group. **(B)** The apoptosis of SH-SY5Y was evaluated by immunofluorescence detection of cleaved caspase-3 (green) and cell nuclei was stained with DAPI (blue) (scale bar: 50 μm). **(C)** The cell death of SH-SY5Y was evaluated by immunofluorescence detection of PI (red) (scale bar: 50 μm).

**Figure 6:**
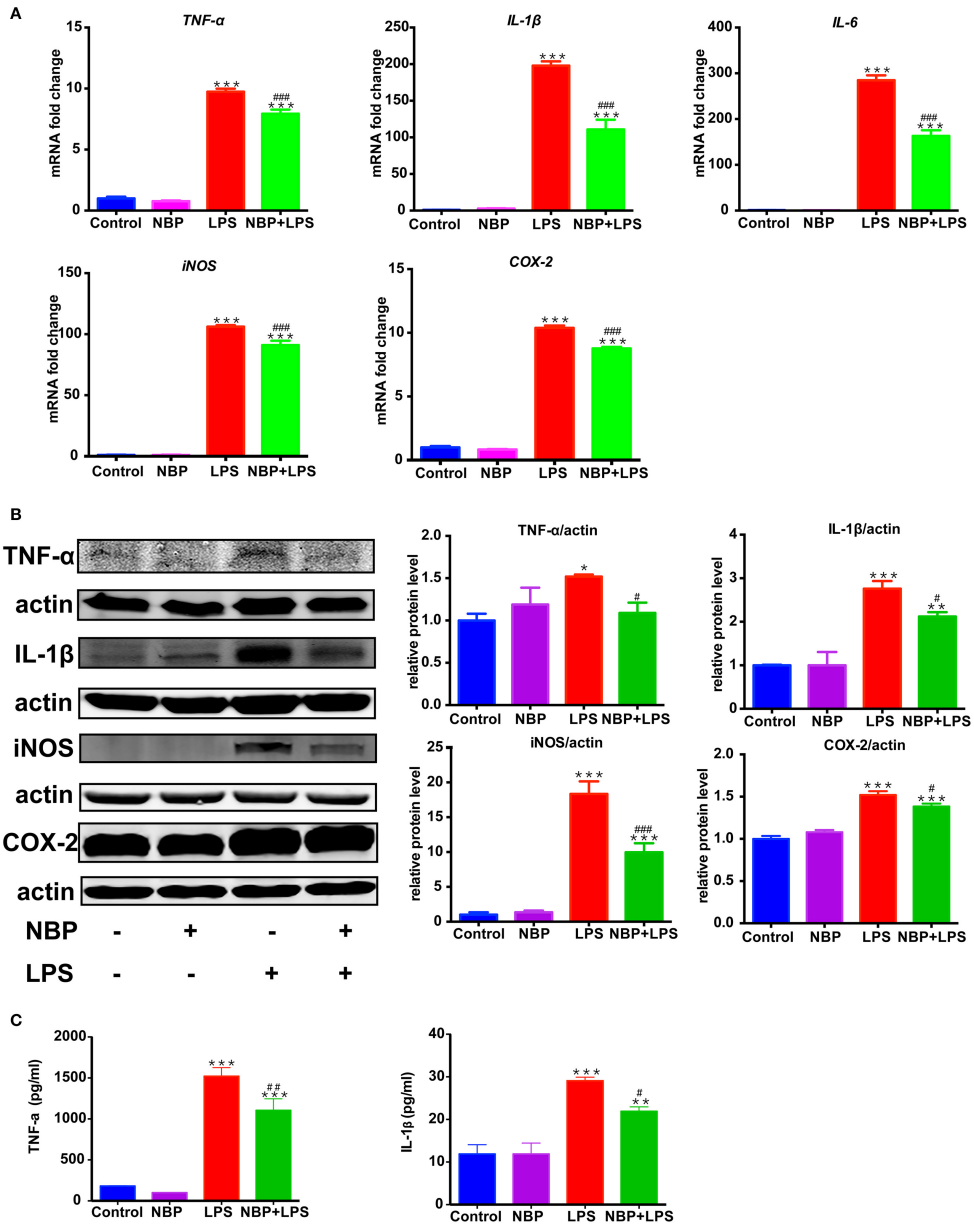
NBP reduced pro-inflammatory molecules expression in LPS-stimulated BV-2 cells. BV-2 cells were pretreated with NBP (0 or 100 μM) for 1 h followed by LPS (0 or 100 ng/ml) for 6 h and total RNA was isolated. To measure cellular protein expression or cytokines level in supernatants, time for LPS treatment was 24 h. **(A)** The mRNA expression of *IL-1*β, *IL-6, TNF-a, iNOS* and *COX-2* was analyzed by RT-PCR and normalized to that of β*-actin*. **(B)** The protein level of TNF-α, IL-1β, iNOS and COX2 was analyzed by Western Blot. **(C)** The level of TNF-α and IL-1β in supernatants was assayed by ELISA kit. All data are presented as means ± SEM (*n* = 3). *p < 0.05, **p < 0.01, ***p < 0.001, compared with the Control group; ^#^*p* < 0.05, ^##^*p* < 0.01, ^###^*p* < 0.001, compared with the LPS group.

**Figure 8:**
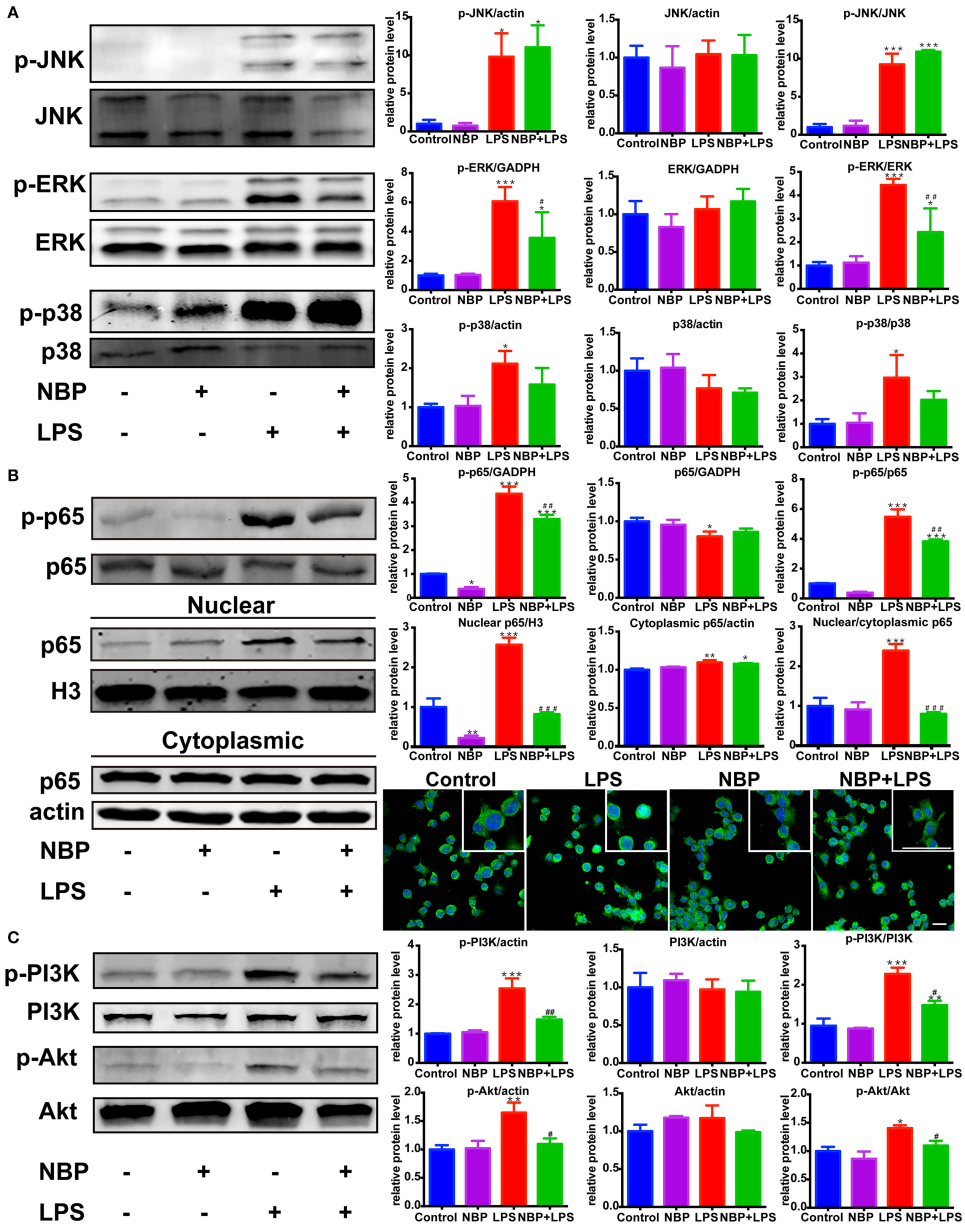
NBP inhibited the activation of ERK, NF-κB and PI3K/Akt pathways in LPS-stimulated BV-2 cells. **(A)** Western Blot assay for MAPK expression. The expression of total JNK/p38/ERK as well as p-JNK/p-p38/p-ERK were analyzed. **(B)** Western Blot assay for NF-κB expression in whole-cell, nuclear and cytoplasmic extracts. The nuclear translocation of p65 was also evaluated by immunofluorescence detection of p65 (green) and cell nuclei was stained with DAPI (blue) (scale bar: 50 μm). **(C)** Western Blot assay for PI3K/Akt expression. The expression of total PI3K/Akt as well as p-PI3K/p-Akt were analyzed. All data are presented as means ± SEM (*n* = 3). *p < 0.05, **p < 0.01, ***p < 0.001, compared with the Control group; ^#^*p* < 0.05, ^##^*p* < 0.01, ^###^*p* < 0.001, compared with the LPS group.

The authors apologize for these errors and state that this does not change the scientific conclusions of the article in any way. The original article has been updated.

