# Supplementary material for: Dl-3-n-Butylphthalide Exerts Dopaminergic Neuroprotection Through Inhibition of Neuroinflammation
Source: Front Aging Neurosci. 2019 Feb 28;11:44. doi: 10.3389/fnagi.2019.00044 (PMC6403182; doi:10.3389/fnagi.2019.00044)
Supplement: Supplementary file 1 [file Table_1.docx]

**Supplementary Data**


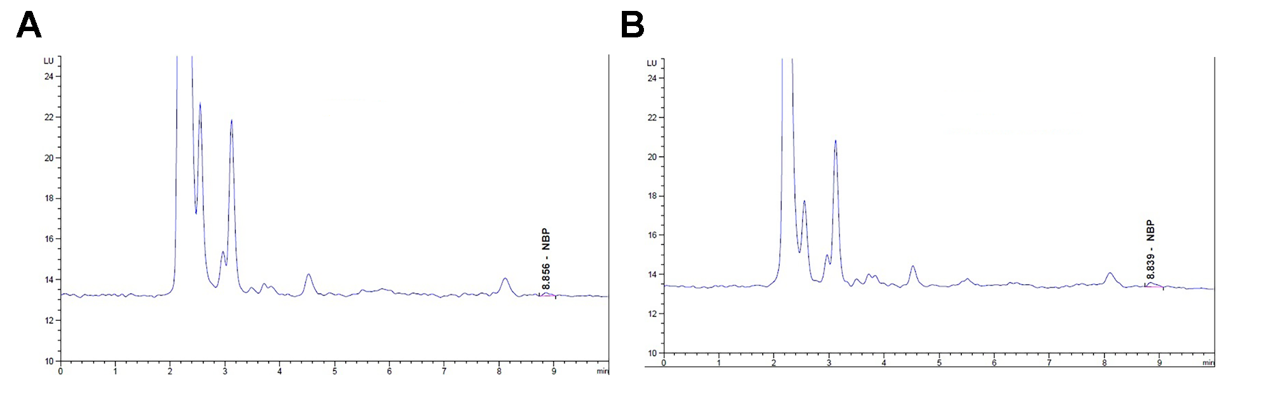


**Fig.S1 Determination of NBP in brain by HPLC.** Mouse brains were collected at 90 min post NBP injection and then processed to examine the delivery of NBP into brain by HPLC. Chromatograms of NBP in the striatum **(A)** and midbrain **(B)**.


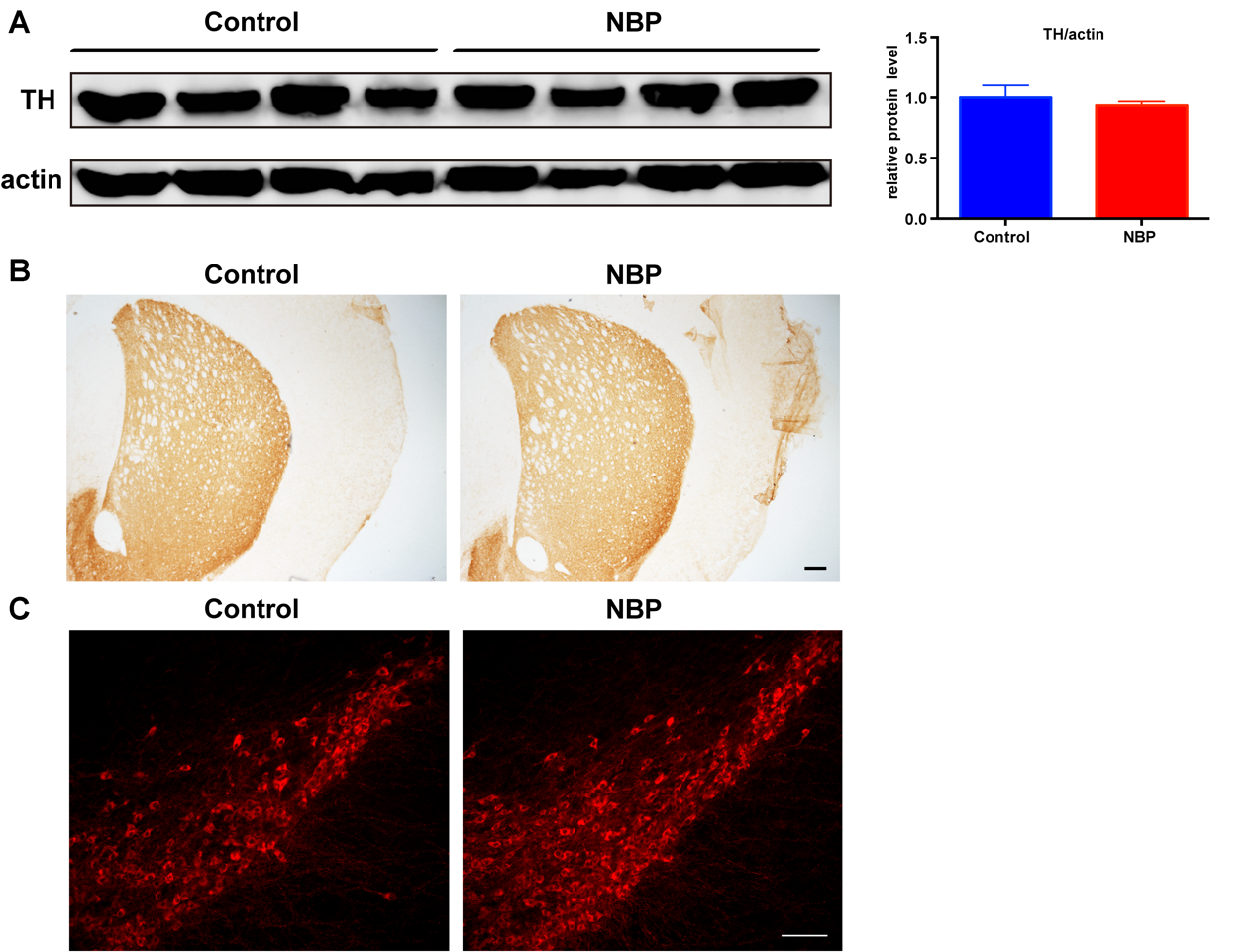


**Fig.S2 NBP alone did not affect the nigrostriatal axis**. **(A)** Western Blot assay of striatal TH protein. Data are presented as means±SEM (n=4). **(B)** Immunohistochemical staining showing TH positive nerve fibers in the striatum. **(C)** Immunofluorescence staining showing TH positive cells in the SN.


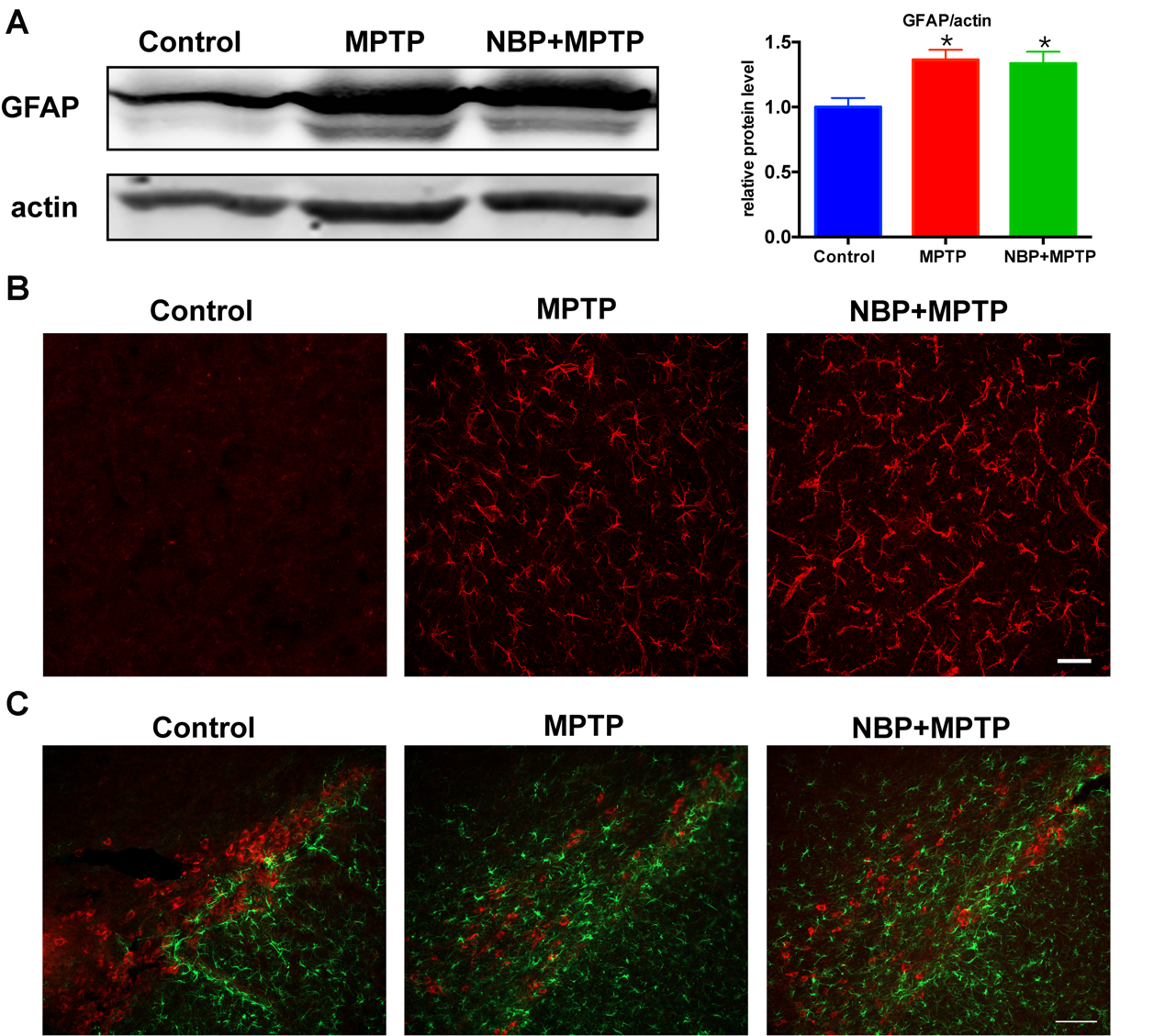


**Fig.S3 NBP did not alter MPTP-induced astrocyte activation in the striatum and SN. (A**) Western Blot analysis of striatal GFAP protein (n=4). Protein band intensity is normalized to actin and is expressed as fold difference relative to the Control group. All data are presented as means±SEM. *p < 0.05, compared with the Control group. (**B)** Immunofluorescence staining staining showing GFAP positive cells in the striatum (scale bar: 50 μm). **(C)** Double immunofluorescence staining of TH (red) and GFAP (green) in the SN (scale bar: 0.1 mm).


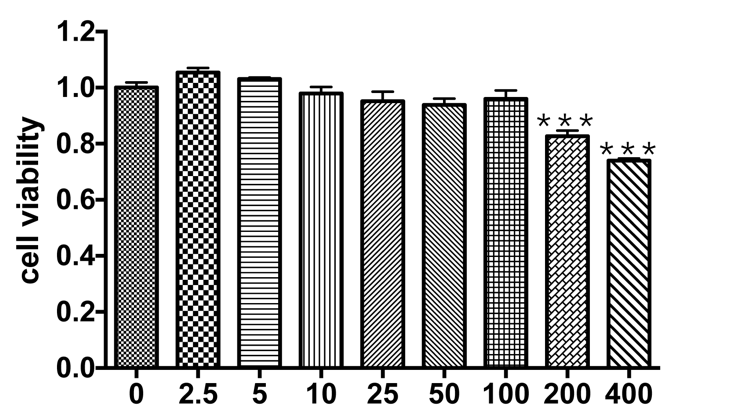


**Fig.S4 NBP (0-100** μ**M) did not affect the** **viability of BV-2 cells**. BV-2 cells were treated with different concentrations of NBP (0-400 μM) for 24 h and cell viability was measured with the CCK8 assay. All data are presented as means±SEM (n=5). ***p < 0.001, compared with the Control group.


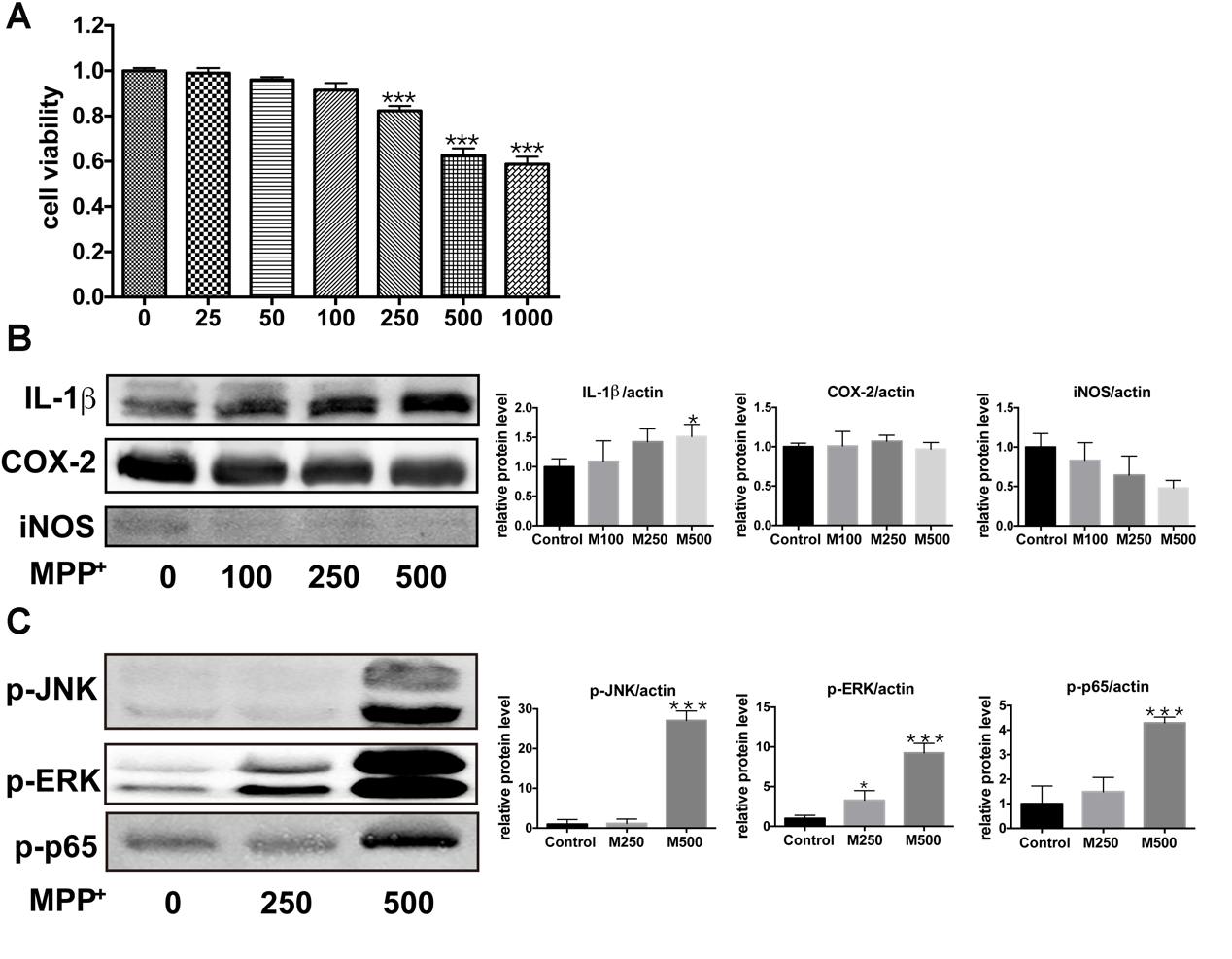


**Fig.S5 The effect of MPP^+^ (0-500** μ**M) on cell** **viability, proinflammatory molecules and signaling pathways in BV-2 cells**. BV-2 cells were treated with different concentrations of MPP^+^ (0-500 μM) for 24 h. **(A)** Cell viability was measured with the CCK8 assay (n=5). **(B)** The protein level of IL-1β, COX-2 and iNOS were analyzed by Western Blot (n=3). **(C)** The phosphorylation of MAPK and p65 were were analyzed by Western Blot (n=3). All data are presented as means±SEM. *p < 0.01, compared with the Control group, ###p < 0.001, compared with the MPP^+^ group.
